# Analysis of Tubular NF Plants in Scotland Indicates That Summer Temperatures and Redox-Sensitive Elements Are Correlated with Membrane Biofouling and Shortened Useful Life

**DOI:** 10.1021/acsestwater.4c00630

**Published:** 2024-10-29

**Authors:** Desislava Filipova Davidkova, Margaret Catherine Graham, David MacLeod, Santiago Romero-Vargas Castrillón, Andrea Joana Correia Semiao

**Affiliations:** † Institute for Infrastructure and Environment, School of Engineering, The University of Edinburgh, William Rankine Building, Thomas Bayes Road, Edinburgh EH9 3FG, United Kingdom; ‡ School of Geosciences, The University of Edinburgh, Crew Building, Alexander Crum Brown Road, Edinburgh EH9 3FF, United Kingdom; § Scottish Water, 31 Henderson Drive, Inverness IV1 1 TR, United Kingdom; ∥ Institute for Materials and Processes, School of Engineering, The University of Edinburgh, Sanderson Building, Robert Stevenson Road, Edinburgh EH9 3FB, United Kingdom

**Keywords:** nanofiltration, water quality, membrane autopsy, full-scale drinking water treatment plant, fouling

## Abstract

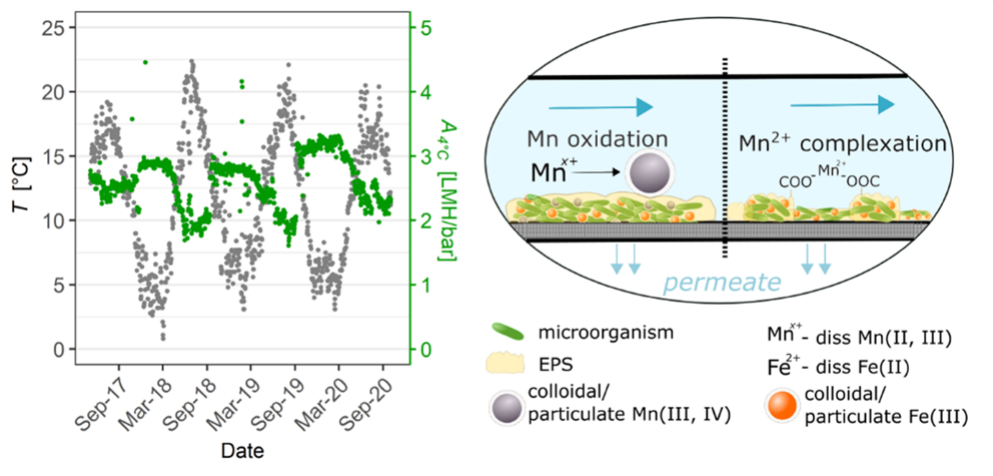

We investigate the effects of seasonal variations in water composition and temperature on the performance of two full-scale drinking water treatment plants in Scotland, equipped with tubular cellulose acetate nanofiltration membranes. Multiple environmental and water quality parameters, recorded over a 4.5-year period, were correlated against membrane permeance, cleaning frequency, and useful life. Membrane autopsies enabled the characterization of the foulant composition. Temporal variations in temperature at plant X led to significant biofouling (manifested by permeance losses of 30–50%, and bacteria detected on the membrane surface) during the summer months, when water temperatures exceeded 20 °C and microbiological activity was highest. Plant Y, in contrast, displayed smaller seasonal variations and was operationally stable without significant fouling. A pronounced increase in manganese and iron (up to 200 and 600 μg/L, respectively) in the lake water at plant X in summer was accompanied by elevated content (∼60 mg/m^2^) of those metals on the membrane surface, which was consistent with lake thermal stratification and metal input from the sediment into the water column. Our work shows that membrane plants in regions supplied by standing surface water bodies, such as plant X, are more vulnerable to biofouling, especially during warmer months.

## Abstract 2

Membrane-based DWTPs supplied by standing water bodies in temperate climates are vulnerable to rising temperatures, due to exacerbated colloidal and biofouling in the summer months.

## 1 Introduction

The capacity of nanofiltration (NF) to remove natural organic matter (NOM), microorganisms, pesticides, and toxic elements renders this technology an important process in potable water production.^1^ NF is applied for surface water and groundwater treatment in temperate and boreal regions such as Scandinavia,^2^ the United Kingdom,^3−5^ France,^6,7^ and The Netherlands,^8,9^ where small- and medium-size membrane drinking water treatment plants (DWTPs) are vital for potable water production in remote communities.^4^ Despite the widespread application of NF, fouling remains a major technical drawback, decreasing membrane useful life and increasing operating costs.^10^

Fouling mechanisms are complex and highly dependent on the raw water composition. The types of foulants on NF membranes treating surface water can include organic (*e.g.*, humic and fulvic substances, polysaccharides),^11^ inorganic (*e.g.*, silica, iron or manganese oxides, metal sulfides),^12^ and biofoulants (*e.g.*, bacteria, algae, protozoa).^13^ Fouling severity can be managed with suitable pretreatment.^7^ However, some membrane plants for the treatment of surface water in Scotland,^3^ northern USA,^14^ Sweden,^15^ and Norway^2^ do not use any form of pretreatment (*e.g.*, flocculation and sedimentation), apart from coarse screening of the feedwater (50–2000 μm), exposing the membranes to foulants such as colloidal organic and inorganic matter as well as bacteria and viruses in the raw water. To combat this problem, DWTPs rely on periodic membrane cleaning.^16^ Often the chemical cleaning agents employed are prescribed by the membrane manufacturer to ensure compatibility with the membrane material. However, these agents might not be best suited to effectively remove membrane foulants specific to a geographic location. Moreover, the concentration of foulants present in the source water can vary seasonally, which further affects the membrane performance and fouling behavior. For example, the operation of DWTPs in temperate regions that abstract water from surface water bodies (*e.g.*, *lochs*, rivers, and reservoirs) is affected by seasonal changes in precipitation and temperature.^17^ These two factors control not only the reservoir water level and *in situ* aquatic processes (*e.g.*, metal cycling and organic matter decomposition)^17,18^ but also larger-scale catchment processes (*e.g.*, runoff and soil weathering),^19^ which determine the DWTP feedwater quality.^6,20,21^ Furthermore, projections of warmer and more frequent extreme weather events in the northern hemisphere, such as prolonged droughts and flash floods,^22,23^ present new water quality challenges for the management of water treatment facilities.^24,25^ To date, however, only a few investigations of full-scale DWTPs^3,8,9^ have been reported. Most NF membrane fouling research is carried out at laboratory scale with simplified artificial feedwater matrices, which fail to capture the effect of variations in real feedwater composition. With climate change exacerbating seasonal variability, more long-term monitoring studies of full-scale membrane facilities and their water sources are needed to better understand temporal water quality changes and their effect on membrane operation.

Here we carry out a comprehensive investigation of the performance and fouling characteristics of two full-scale membrane DWTPs in Scotland during a 4.5-year operation period. Long-term data analysis of multiple environmental and water quality parameters (water composition and temperature, pH, TOC, coliforms, *etc.*) together with membrane autopsy to characterize the composition of foulant layers enabled us to identify the main determinants of fouling in two DWTPs treating distinct surface waters. Our study provides a framework for future DWTP investigations along with practical insight into the influence of changing environmental conditions on membrane performance and useful life.

## 2 Materials and Methods

**Figure 1:**
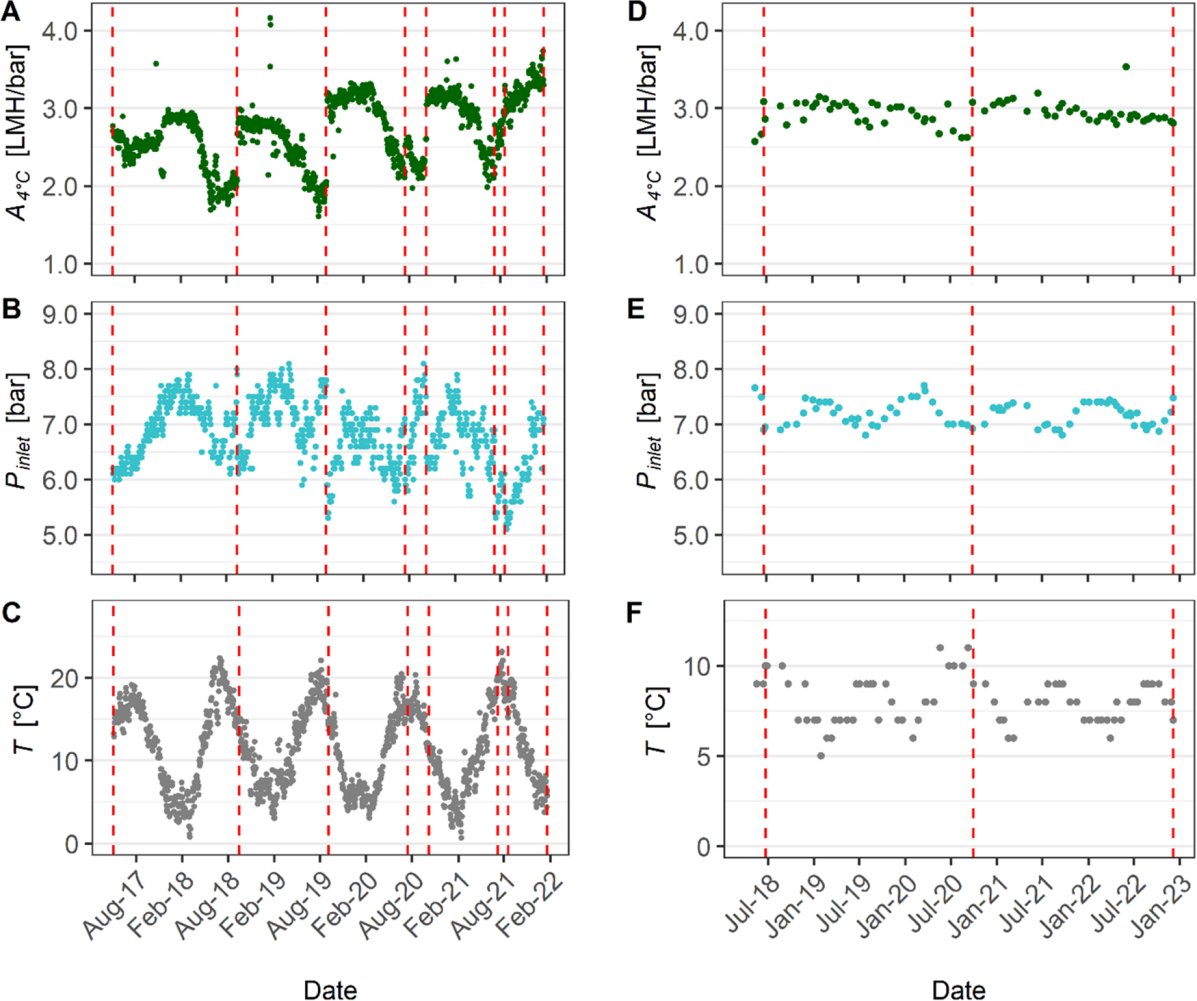
Membrane operating parameters over time for plant X (A–C) and plant Y (D–F) including permeance (*A*_4°C_), inlet pressure (*P*_inlet_), and temperature (*T*). The red dashed lines indicate dates of the membrane change. Note: the scales of the *y*-axis in (C, F) differ.

Two full-scale DWTPs, operated by the local public drinking water provider Scottish Water, were chosen to investigate and compare the NF membrane performance in different areas of Scotland. Plant X was located on an island off the west coast of Scotland, while plant Y was situated in the Scottish Highlands, approximately 300 km north of plant X. Both DWTPs used C10 modules (PCI membranes, Poland) equipped with 72 tubular cellulose acetate CA202 membranes connected in series, and with a total membrane area of 10.4 m^2^ per module. Plant X had 35 modules, and plant Y had 24 modules, giving a total membrane area of 364 and 249.6 m^2^, respectively. The membranes were operated in cross-flow mode (cross-flow velocity ∼0.6 m/s) at a constant permeate flow rate set to ∼6.3 m^3^/h and ∼4.2 m^3^/h for plants X and Y, respectively (Figure S1A,D), yielding a nominal permeate flux of ∼17 L m^–2^ h^–1^. This was achieved by adjusting the inlet pressure (*P*_inlet_), with an average *P*_inlet_ of 7.2–7.5 bar (Figure 1B,E). Both DWTPs used surface water as feedwater. However, the source for plant X was a shallow (mean depth = 4.5 m) low-altitude lake, while plant Y was supplied with freshwater from a stream (Table S1). Mechanical cleaning by foam ball scouring of the membrane tubes was carried out for 5–10 min every 4–6 h, as per the manufacturer’s recommendations. Regular chemical cleaning (0.2% w/v citric acid recirculation for 1 h at design total feed flow rate of 9.6 m^3^/h and 5.6 m^3^/h for plant X and Y, respectively, with equivalent velocities of 0.60 and 0.54 m/s) was performed routinely every 3 to 4 months with additional cleaning undertaken to alleviate high applied *P*_inlet_ when needed, due to fouling build-up on the membrane.

Past records of the permeate flow rate, inlet and outlet pressures, and temperature, covering a 4.5-year period, from May 2017 to January 2022 for plant X, and between April 2018 and December 2022 for plant Y, were provided by Scottish Water to assess the membrane performance. To allow for comparison between the two plants over time, a temperature-corrected (to *T* = 4 °C) permeance (measured in L m^–2^ h^–1^ bar^–1^, henceforth LMH/bar) was calculated to account for the effects of temperature on water viscosity (as done in previous studies^8,26^) using the following equation:^27^
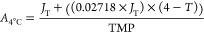
1where *J*_T_ is the permeate flux [L m^–2^ h^–1^] at temperature *T* [°C];  is the transmembrane pressure difference [bar]; and *P*_inlet_, *P*_outlet_, and *P*_permeate_ are the pressures measured at the inlet, outlet, and permeate side of the membrane, respectively. The osmotic pressure was neglected, as it does not exceed 0.1 bar for fresh waters (see Section S.1). The feedwater quality parameters were measured in Scottish Water Laboratories using analytical protocols, facilities, and materials approved by the United Kingdom Accreditation Service (UKAS). Experimental protocols to determine total organic carbon (TOC), elemental composition (Al, Fe, Mn), *Escherichia coli*, coliform colony forming units (CFUs) and total cell counts (TCC) are explained in detail in the Supporting Information (see Section S.2). Statistical analysis protocols using Spearman’s rank correlation test are also explained in Section S.2. Membrane autopsy and characterization protocols of membrane foulants (extracted TOC and protein content; elemental analysis; characterization of foulants by fluorescence excitation-emission matrix (FEEM) spectrophotometry; and scanning electron microscopy (SEM) imaging) are given in Section S.3. Characterization of membrane transport properties is given in Section S.4.

## 3 Results and Discussion

### 3.1 NF Membrane Performance Decline with High Summer Temperatures

Evaluation of the archival data showed that the permeance (*A*_4°C_) decline, membrane replacement, and membrane cleaning frequencies differed between the two DWTPs (Figures 1, S1, and S2). The average membrane lifespan of 2 years and 3 months at plant Y was notably higher than that at plant X (∼1 year) and closer to the optimal CA202 membrane operational time of 3 to 5 years, as indicated by the manufacturer.^28^ The *A*_4°C_ loss between membrane replacements for plant Y was between 9 and 15% (Figure 1D). The citric acid chemical cleaning at the plant was carried out on average every 49 days between June 2018 and September 2020 (Figure S2B), and every 72 days thereafter (likely due to the relatively small decline in *A*_4°C_ over the post-September 2020 time period). This cleaning method, as shown in Figure S2B, maintained the membrane permeability throughout the membrane lifespan.

Notably, *A*_4°C_ at plant X decreased by ca. 30–50% within a 1-year period between membrane changes (*e.g.*, from September 2018 to September 2019, from September 2019 to July 2020, and from October 2020 to July 2021) (Figure 1A). The membrane permeance at plant X exhibited strong seasonal dependence: stable in late autumn and winter (between October and February) with a significant decline during spring, reaching the lowest *A*_4°C_ values in summer and early autumn (Figure 1A). The average membrane cleaning frequency (Figure S2A) for plant X was 102 days. The citric acid cleaning strategy was not effective in recovering *A*_4°C_ in the months of significant permeance loss (March to September), indicating that alternative cleaning strategies are needed at plant X. Membrane substitution was required for plant X with a higher frequency compared to plant Y, *i.e*., 8 substitutions *vs* 3 substitutions, respectively, and most substitutions occurred during the summer/autumn seasons. The data analysis showed a strong negative correlation between *A*_4°C_ and temperature (*r* = −0.74, Figures S3A and 1A,C) indicating that *T* is an important covariate in plant X. The negative correlation between *T* and *A*_4°C_ suggests that fouling is responsible for the observed trend. The lower maximum feedwater temperature during summer at plant Y (*T*_max_ = 10 °C) compared to plant X (*T*_max_ = 20 °C) (Figure 1C,F) can be linked to the geographical location and feedwater source type. Meteorological data from the two locations show higher average ambient temperatures throughout the whole year at location X compared to that at location Y (Figure S4). Furthermore, the shallow low-altitude standing lake water at plant X will be subject to less mixing compared to the flowing stream at plant Y. Therefore, plant X water is predisposed to more heating by solar radiation. Next, we examined the feedwater composition throughout the year in order to identify the potential causes for poor membrane performance.

### 3.2 Feedwater Quality

**Figure 2:**
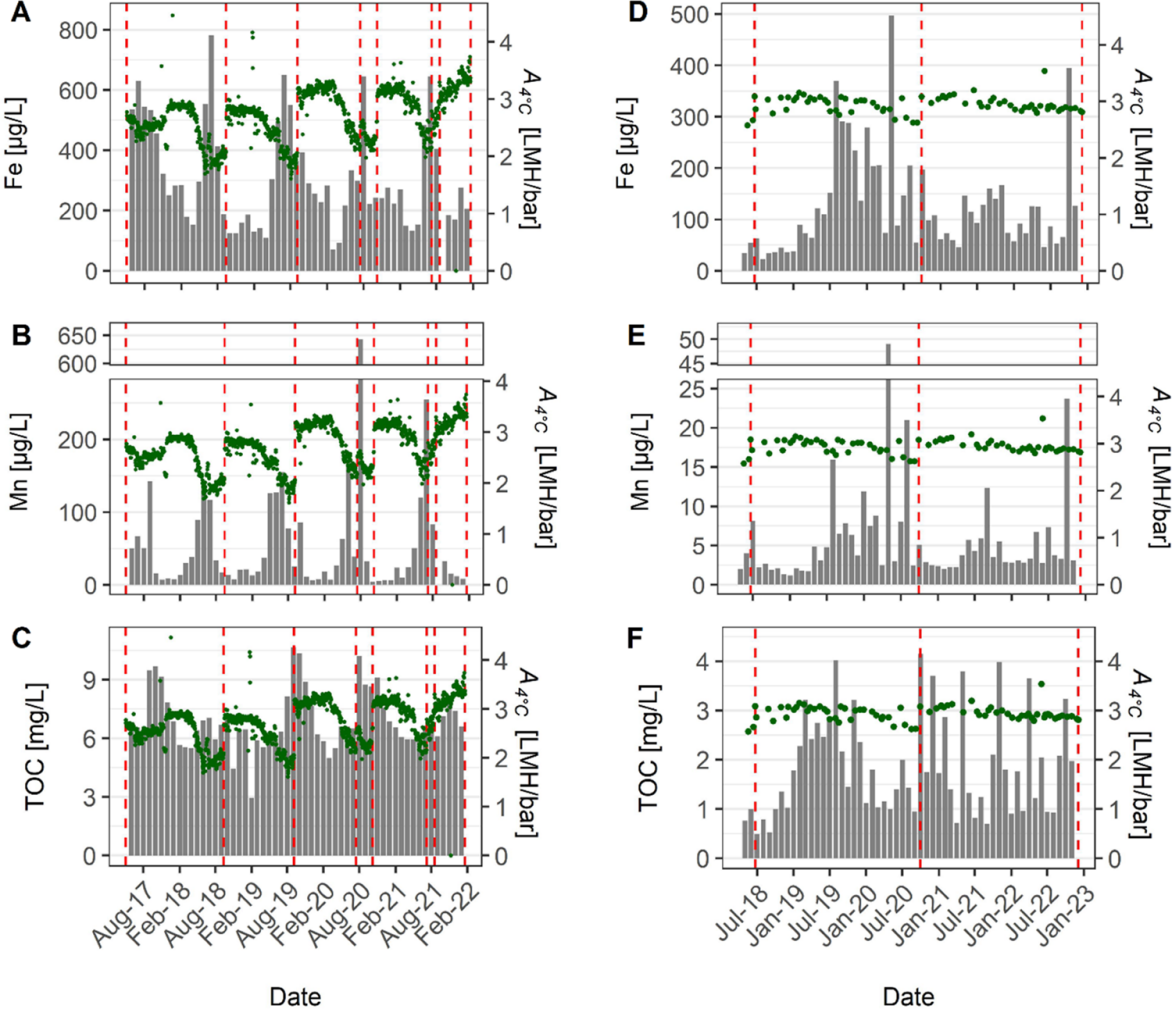
Monthly averaged feedwater quality parameters (bar plot) *vs* time overlaid with permeance (*A*_4°C_—scatter plot; right *y*-axis) for plant X (A–C) and plant Y (D–F). The red dashed vertical lines indicate dates of the membrane change. Note: the scales of the *y*-axes differ.

Feedwater quality parameters of the two DWTPs are summarized in Table S2, and monthly averaged values of selected parameters plotted over time are presented in Figures 2 and S5. Both DWTPs had a similar neutral-alkaline pH ranging from 7 to 8 (Table S2). The feedwater for plant Y had low feedwater conductivity (127 ± 24 μS/cm) typical of Scottish surface waters.^5,25,29^ Conductivity records for plant X were not available, but these are expected to be similar to those of Scottish *lochs* (77–230 μS/cm).^5,25,29^ In terms of microbiological parameters, the TCC at plant X was about 5 times higher than that at plant Y (Table S2). Although TCC at plant X showed only moderate correlation with temperature (*r* = 0.4, Figure S3A) and no discernible seasonal trend (Figure S5D), the warmer feedwater—reaching 2 times higher temperatures in summer compared to plant Y (Figure 1C,F)—is consistent with the higher biological activity at plant X compared to plant Y.

#### 3.2.1 Elevated TOC and Al in Autumn due to Catchment Processes

The high TOC content at plant X (7.0 ± 1.6 mg C/L, Table S2) compared with the low concentrations at plant Y (1.9 ± 1.9 mg C/L) can be linked to the elevated summer temperatures and microbial activity. Notably, the peak in TOC concentrations in the source water for plant X occurs in late summer to autumn (Figure 2C). Similar seasonal trends have been observed in studies investigating the NOM precursors for trihalomethane formation in Scottish water treatment and supply facilities.^25,29^ This autumnal peak in TOC content can be explained by local carbon cycling and hydrology of standing water bodies.^30^ In spring-summer, the lake water level drops and the soil water content also decreases due to evaporation and reduced rainfall (Figure S4). Enhanced aerobic microbial activity increases the dissolved organic carbon in the freshly exposed dry soil layers that would otherwise be submerged or waterlogged.^31^ These organic compounds enter the water column during heavy autumnal rainfall and runoff events (Figure S4), contributing to higher TOC concentrations between August and November.^19,21,29,30^ Increase in precipitation has been linked to more significant organic carbon mobilization from soil to freshwater in Scandinavian countries as well.^32^ Aluminum concentrations in the water to plant X also experience an increase during autumn and winter (Figure S5A). Al is known to strongly bind to NOM.^33^ This suggests that Al transport may be linked to NOM influx (see Figure S3A; *r*_Al∼TOC_ = 0.37) from the catchment during autumnal rainfall and runoff events rather than elemental remobilization within the aquatic environment and sediment.^19^

#### 3.2.2 Elevated Fe and Mn in Summer due to Metal Input from the Sediment

The most abundant metal measured in the source water for both DWTPs was Fe (Table S2). There were, however, differences between locations: plant X had more than 2 times higher average Fe concentrations and an order of magnitude higher Mn feed concentration than plant Y (Table S2). The concentrations of Fe and Mn also varied seasonally (Figure 2A,B,D,E). The Fe and Mn concentrations in the source water at plant X peaked in summer (between May and August), exceeding the consumer drinking water standards (prescribed concentration or value (PCV) = 200 μg Fe/L and 50 μg Mn/L^34^). These concentrations decreased in winter to ∼200 μg Fe/L and <10 μg Mn/L (Figure 2A,B). Significant seasonality in Mn and Fe concentrations was only observed at plant X, where feedwater is sourced from a lake. The seasonal trend was statistically supported by a strong correlation between the metals’ feed concentrations and temperature (*r* > 0.58, Figure S3A). In contrast to the autumn peak in TOC and Al concentrations, which are most likely controlled by catchment processes, the seasonality observed for Fe and Mn is attributed to *in situ* metal cycling within the lake water column and sediment.^18,35^ Although there was no dissolved oxygen (DO) data available for the feedwater, it is well-known that without mixing the DO content in standing water bodies (*e.g.*, reservoirs and lakes) decreases with increasing water depth.^17,36^ These conditions are exacerbated during prolonged dry summer periods when there is little advective mixing (*e.g.*, by rainfall and wind) within the water column, and elevated microbial activity depletes DO.^37^ The resulting low DO conditions close to the water-sediment interface promote the release of Mn^2+^ and Fe^2+^ into the water column through reductive dissolution of Mn and Fe (hydr)oxide minerals in the sediment.^18,37−39^ Low DO conditions are consistent with data for plant X, where high summer feedwater temperatures (Figure 1C) are responsible for the elevated TCC content (Table S2) and higher microbial activity, which in turn drive a faster depletion of the DO. Furthermore, low DO has been documented for lakes (including shallow lakes^40−43^) in temperate regions (in the Northern hemisphere) that experience thermal stratification during the summer, followed by reversal during the autumnal turnover.^17,36,37,39,44^ Lower Mn and Fe concentrations in autumn and winter (Figure 2A,B) are thus consistent with higher rainfall (Figure S4) and, consequently, DO in the water column. Higher DO concentrations brought about by the mechanical mixing of the water column in autumn lead to the formation of Mn and Fe (hydr)oxides that are subsequently removed through gravitational settling, thereby reducing their concentrations in the water column.^36,39,44^ Although there are strong similarities in Mn and Fe behavior, the difference in oxidation rate between these two elements (slower oxidation rate for Mn than Fe) may mean that during the summer concentration peak, they are present within the loch waters in different forms. Previous experiments testing the release of Fe and Mn from sediment cores under limited DO conditions in low light have confirmed that over a 24-day period, all of the measured Mn in the water column above the core was in soluble form (*e.g.*, Mn_(aq)_^2+^), while the majority of the Fe was in suspended colloidal phase (*e.g.*, Fe(OH)_3_).^18^ Similar observations have been made in a thermally stratified lake in the USA, where during the summer anoxic period all measured Mn in the water column was in soluble form, while Fe was present in particulate form as well.^44^ Reported values from field and laboratory experiments for soluble iron half-time (*t*_0.5_), defined as the time required for half of the initial mass of soluble Fe to oxidize, consistently show that even under abiotic conditions (*e.g.*, without microbial activity) *t*_0.5_ ranges from minutes up to 2 days.^36,38^ However, the wide range of *t*_0.5_ reported for Mn—from 1 day up to more than a 1 month—was explained by the significant catalytic effect that Mn-oxidizing organisms can have on the reaction.^36,45^ Considering the high TCC recorded in the feedwater to plant X, biological processes also need to be taken into account when discussing the fate of redox-sensitive elements like Mn and Fe within the DWTP and on the membrane surface. We discuss the connection between the biogeochemistry of these metals, feedwater quality, and membrane performance in Section 3.4.

In contrast to plant X, no strong seasonal dependency or breach of water quality standards was observed at plant Y (Figures 2D–F and S5E–H), suggesting stable operation of the plant throughout the year (see Figure 1D). Considering that the feedwater source is a flowing stream, the continuous mixing hinders thermal stratification and pronounced variation in feedwater composition. This is evident from the overall lower and less variable feedwater temperature throughout the year at plant Y compared to plant X (Figure 1F). However, there is a peak in *E. coli* and coliform CFUs in summer 2021 (Figure S5F,G), supported by moderate correlations between *T* and the two types of bacteria (*r* ∼ 0.3–0.4, Figure S3B), suggesting that rising temperatures could promote higher microbial activity and cause future biofouling problems even at DWTPs supplied by rivers.^46^

Based on the data for plants X and Y, the water quality temporal analysis points out that standing water bodies (such as that in plant X) are predisposed to elevated feedwater temperatures, microbial activity, and metal (Mn and Fe) concentrations in summer compared to well-mixed flowing streams (plant Y), which in turn leads to permeance loss in summer.

### 3.3 Foulant Characteristics of NF Membranes

#### 3.3.1 Surface Imaging of the NF Membranes

To examine the qualitative appearance of membrane coupons, individual membrane tubes were cut lengthwise to reveal the active layer color and morphology after fouling. All membrane tubes collected from plant Y exhibited few regions of brown coloration (Figure S6B), the membranes being qualitatively similar to the pristine membrane (Figure S6C). The SEM cross-sectional images further confirmed that the surface of the membranes from plant Y appeared smooth, similar to the pristine membrane (Figure S6B,C).

The tubes from plant X, on the other hand, had different levels of brown coloration. Based on the initial visual inspection and brown color coverage, these were grouped as high, mid, and low fouling severity (Figure S6A). The location of the tubes within the membrane installation was not noted, so it was not possible to attribute the different extent of fouling to specific parts of the membrane channel (*e.g.*, inlet *vs* retentate section of the membrane train). The SEM imaging of membrane tubes from plant X of high fouling severity confirmed that the surface appeared rougher compared with the sample from plant Y and the pristine membrane (Figure S6A).

#### 3.3.2 Organic and Biological Foulants

The organic and biological foulants on the membrane surface were quantified *via* TOC and protein analysis, respectively. The TOC and protein concentrations measured on the membrane samples from plant X varied between tubes of different fouling severity (as determined by the membrane coloration), with heavily fouled membranes having higher protein (5.91 ± 5.53 μg/cm^2^) and TOC (8.77 ± 3.45 μg/cm^2^) content compared to the mid and low levels of fouling (Table 1). Overall, the concentrations were on the lower end of those reported in previous NF and RO membrane autopsy studies, which ranged from 0.8 to 47 μg/cm^2^ protein^9^ and 5–150 μg/cm^2^ TOC.^8^ It should be noted that the extraction procedure applied here, using UP water and mechanical mixing, is an incomplete extraction method and that some of the coloration on the membrane surface remained. Therefore, the obtained TOC and protein contents reflect the loosely bound foulant and, hence, the total organic and biological foulants accumulated on the membrane surface is likely to be higher than the values reported in Table 1.

**Table 1:** Inorganic and Bio-Organic Composition of Membrane Deposits^a^

inorganic composition [mg/m^2^]					
	plant X			plant Y	pristine
	high	mid	low		
Fe	62.8 ± 16.9	20.2 ± 11.4	16.3 ± 17.9	7.7 ± 5.9	3.4 ± 3.1
Mn	62.3 ± 15.6	8.1 ± 3.8	8.3 ± 7.6	0.3 ± 0.8	0.1 ± 0.07
Al	7.3 ± 2.2	1.9 ± 1.1	0.7 ± 1.1	2.1 ± 1.6	ND
Mg	4.7 ± 1.3	2.1 ± 0.7	1.5 ± 0.9	4.3 ± 1.9	0.5 ± 0.7
Ca	4.4 ± 1.3	2.0 ± 0.9	1.4 ± 0.5	21.2 ± 8.1	2.2 ± 1.7
Na	43.7 ± 29.9	33.8 ± 26.1	27.0 ± 22.3	97.5 ± 70.1	31.9 ± 33.9

organic composition [μg/cm^2^]					
	plant X			plant Y	pristine
	high	mid	low		
protein	5.91 ± 5.53	1.33 ± 0.94	2.37 ± 1.62	1.76 ± 3.89	0.16 ± 0.83
TOC	8.77 ± 3.45	5.35 ± 6.38	2.29 ± 0.79	4.06 ± 0.31	2.75 ± 0.75

a Each entry shows the mean (± sd) calculated from three replicate membranes of each type (high, mid, low fouling) for plant X, and 9 replicate membranes for plant Y; nd—not detected.

**Figure 3:**
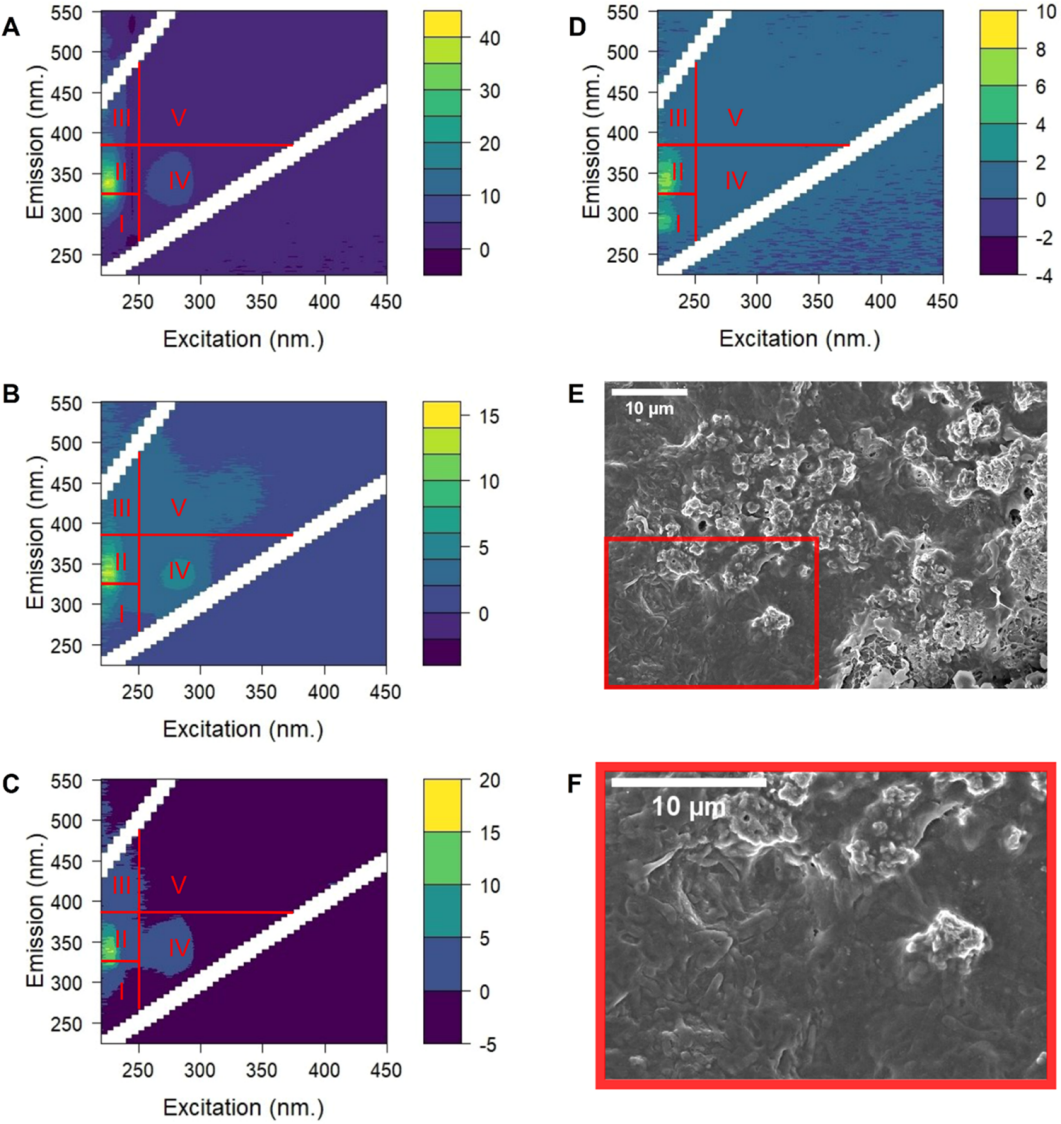
FEEM analysis of UP water-extracted foulant deposits on membranes from plant X (A—high; B—mid; C—low fouling severity) and plant Y (D); SEM images of deposits on tubular membranes from plant X showing rod-shaped bacteria on the membrane surface at low magnification (E) and at high magnification of the area indicated in red on image E (F); EEM regions: I—aromatic protein I; II—aromatic protein II; III—fulvic acid-like substances; IV—soluble microbial byproducts; V—humic acid-like substances. Note: the scales of the *y*-axis differ between A–C and D; the fluorescence intensity is given in arbitrary units (a.u.).

The loosely bound extracted foulant samples were analyzed by fluorescence excitation-emission matrix (FEEM) spectrophotometry to provide further information about the composition and relative abundance of the organic and biological substances extracted from the membrane surface. As shown in Figure 3A–C, the peak fluorescence intensities of the samples from plant X were at regions I, II, and IV, which are indicative of the presence of protein and microbial byproducts.^47^ Similarly, plant Y also shows fluorescence signals consistent with the presence of proteins and microbial byproducts, though to a lesser extent than plant X (Figure 3D). These results suggest that biofouling could be more significant than NOM fouling, which is linked to fluorescence peaks occurring at higher wavelengths located in regions III (fulvic acid-like substances) and V (humic acid-like substances). The humic-associated peak (region V) was observed only in plant X (Figure 3B) and was largely absent in the plant Y data, whereas the fulvic acid fraction was observed in all samples, though at a lower intensity compared to biofoulants. Besides the presence of proteins, SEM images of the heavily fouled membrane tubes from plant X also confirmed the presence of rod-shaped bacteria on the surface, corroborating the findings from the qualitative fluorescence analysis (Figure 3E,F). Notably, the fluorescence intensity is highest for the heavily fouled membranes from plant X with maximum values of 40 au and lower for the less fouled membranes from plants X and Y (up to 10–20 au) (Figure 3A,D). Overall, the qualitative and quantitative bio-organic analyses support the view that biofouling is more significant than NOM fouling in plants X and Y.

#### 3.3.3 Inorganic Foulants

The inorganic composition of the decommissioned membranes was quantified after heated acid (aqua regia) digestion by inductively coupled plasma-mass spectrometry (ICP-MS) analysis (see full details in Section S.3 in the SI). The results suggest that the dominant metals accumulated on membranes from plant X are iron and manganese with average concentrations for both elements varying between 8 and 63 mg/m^2^ depending on the membrane tube (Table 1). Aluminum, magnesium, and calcium were less abundant (<10 mg/m^2^) compared to Fe and Mn on the membrane from plant X. Overall, the inorganic content on the membrane from plant Y was lower compared to plant X for all elements, except for calcium (Table 1). Since no data on Ca concentrations in the feed were available, it was not possible to explain the source or effect of this element on the performance of the membrane at plant Y. However, the measured concentrations are consistent with those recorded for spiral-wound membranes from surface water treatment facilities in The Netherlands,^9^ which reported 15–20 mg Ca/m^2^. The elevated concentrations of sodium observed for all samples, including the pristine membrane, are most likely due to the sodium metabisulfite preservative used for storage; therefore, the values do not represent the sodium accumulated during operation.

Notably, Fe and Mn content at plant X was linked to the coloration of the membrane active layer (indicative of fouling severity). This was determined from the initial visual inspection of the membranes, with the highest average concentrations of ∼63 mg/m^2^ for the heavily fouled tubes (Table 1). The mean iron content for the membranes with mid- and low fouling levels was between 16.3 and 20.2 mg/m^2^, while the manganese concentrations were around 8 mg/m^2^. The Fe and Mn content on the membranes from plant Y was lower than that at plant X, further suggesting these two metals contribute to the coloration on the active layer side of the membranes in plant X (Figure S6). Indeed, Fe and Mn on the surface of a heavily fouled membrane tube from plant X were confirmed using energy-dispersive X-ray spectroscopy (EDX) (Figures S7 and Table S3). The point-sampled spectra show regions with elevated oxygen, moderate manganese, and iron and low carbon levels (Figure S7 and Table S3). Considering that the membrane DWTPs operate with oxygenated surface water at near-neutral pH (Table S2), the metals in the feed, which are ultimately deposited on the membrane surface as foulants, could be present in their soluble form or as oxidized colloids or particulates.^38,48^ Iron-containing minerals inside and on the membrane surface have been found in previous autopsy studies.^8,26^ Examples include surface water treatment by hollow-fiber UF in China, where membranes acquired mahogany coloration,^26^ similar to that observed in the current study (Figure S6), and in contrast to the black deposits on the spiral-wound NF membranes from a groundwater (anoxic) treatment facility in The Netherlands.^8^ This is again attributed to the geochemical cycling of redox-sensitive metals in aquatic systems and their tendency to form red-brown iron (oxy)(hydro)oxide species in oxidizing conditions and black precipitates in reducing sulfur-rich environments.^38^

### 3.4 Dominant Foulants and Fouling Mechanisms

The initial assessment of the operating parameters indicated stable membrane performance at plant Y throughout the year, in comparison to persistent permeance loss and frequent (yearly) membrane change at plant X. The correlation analysis between the temperature-corrected membrane permeance (*A*_4°C_) and water quality parameters at plant X, revealed negative correlations between *A*_4°C_ and Mn (*r* = −0.66), *A*_4°C_ and Fe (*r* = −0.22), and *A*_4°C_ and TCC (*r* = −0.63) (Figure S3A). The behavior of these water constituents was discussed in the context of natural thermally stratified freshwater reservoirs^36−39,44^ (Section 3.2). Although other studies have investigated the behavior of Mn and Fe in the feedwater,^20,36,44^ on the membrane surface^49,50^ and in the drinking water distribution network^51−53^ separately, here we provide a conceptual model of the potential pathways for the main water constituents (Mn, Fe, microorganisms), as well as their interactions and environmental controls.

#### 3.4.1 Fe Cycling, Transport, and Interactions at the Membrane Surface

**Figure 4:**
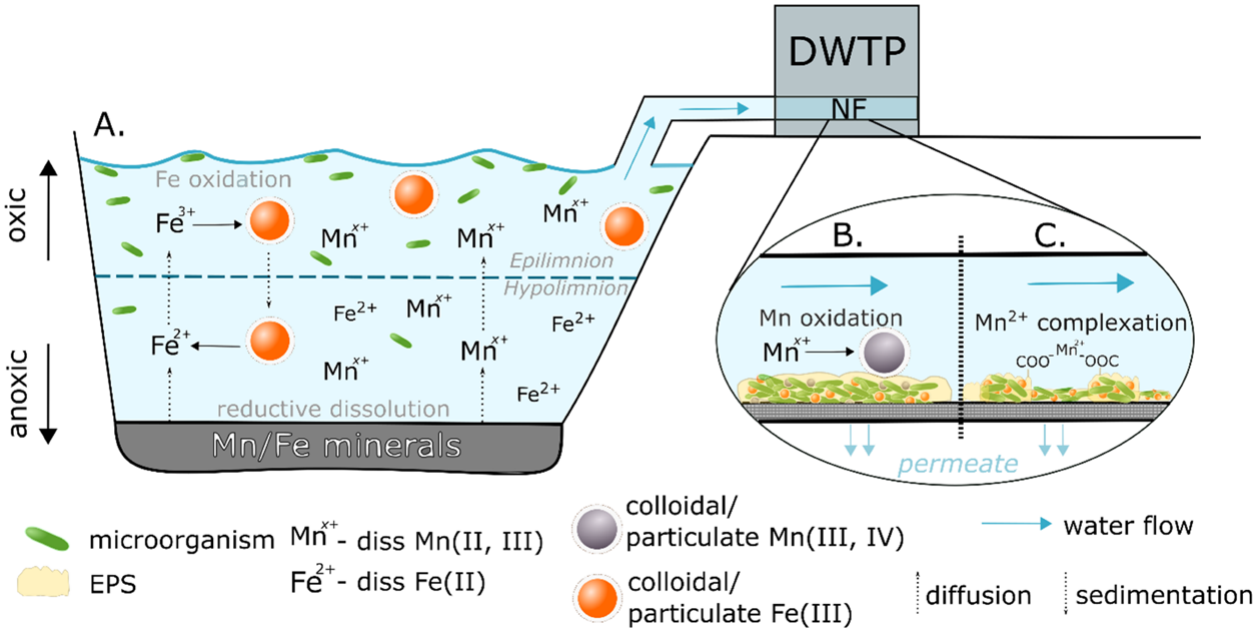
Schematic diagram of the proposed processes involved in Mn and Fe biogeochemical cycling in a thermally stratified lake (A) and on the surface of a biofouled membrane (B, C). diss—dissolved; EPS—extracellular polymeric substances.

The elevated summer Fe and Mn concentrations (Figure 2A,B), explained by reductive dissolution at the water-sediment interface under low DO conditions, suggest that in the hypolimnion, both elements will be in their dissolved reduced form (Figure 4). However, as they diffuse upward, reaching the epilimnion (the top oxygen-saturated part of the water column), they oxidize.^38^ Any large Fe/Mn (oxy)(hydr)oxides would undergo gravitational settling, while the nonsettling colloidal and soluble forms would remain suspended in the water column, eventually becoming entrained by the DWTP feed stream. Dissolved Fe undergoes rapid oxidation (*t*_0.5_ of up to 48 h^36,38^) and, therefore, is predominantly present in its colloidal/particulate form when it reaches the membrane. Upon deposition, the iron colloids would become embedded in the biofilm present on the membrane surface (Figure 3).^49,50^ A similar mechanism operates in gravity-driven membrane filtration, which uses a flat-sheet UF membrane and allows the formation of a biofilm layer as an extra barrier to foulants.^49^ Similarly, previous laboratory membrane filtration experiments using alginate, a model foulant that mimics EPS produced by microorganisms, have proposed that the formation of nanosized FeOOH-alginate aggregates (within a Fe–Ca-alginate system) exacerbates fouling and contributes to a more compact layer compared to that observed within a Ca-alginate system.^50^

#### 3.4.2 Mn Cycling, Transport, and Interactions at the Membrane Surface

The behavior of Mn within the DWTP can differ significantly from that of Fe due to its slower oxidation rate (*t*_0.5_ ranging from 1 day up to several months^36,45^). Some studies have reported dissolved Mn to be dominant even in the epilimnion,^36,44,54^ while other papers have reported waters with over 90% of Mn in colloidal/particulate form (>0.2 μm).^55^ Apart from different size fractions (dissolved, colloidal, and particulate), Mn can also exist in three oxidation states: Mn(II), Mn(III), and Mn(IV).^38,55−57^ In its dissolved ionic form, Mn(II) and Mn(III) would be poorly rejected by loose nanofiltration membranes such as the CA202 (*R* = 6.2 ± 1.0%; for 0.3 mM CaCl_2_, see Table S4 and Section S.4) and would not cause major performance issues at low concentrations on its own.^58^ However, the Mn content accumulated on the heavily fouled membranes obtained from plant X was comparable to that of iron (∼63 mg/m^2^) (Table 1), even though the feedwater concentration of Mn was around 3 times lower than Fe in summer (Figure 2A,B), suggesting that there are additional mechanisms, discussed below, promoting Mn deposition at the membrane surface.

##### 3.4.2.1 Effect of Abiotic Interactions (NOM and Mineral Surfaces)

Previous studies under abiotic conditions have reported that NOM and mineral surfaces (*e.g.*, suspended inorganic colloids) play an important role in Mn transport and speciation.^36,54−56^ As already mentioned, due to the slower oxidation rate of Mn compared to Fe, the dissolved Mn often coexists with Fe(III) colloids in oxic surface waters (Figure 4A). Mineral surfaces have a catalytic effect on Mn(II) oxidation reactions.^56,57^ Adsorption of Mn(II) and O_2_ on the mineral surface increases the local concentration of the sorbates and promotes the formation of Mn (III, IV) (oxy)(hydr)oxides.^56,57^ NOM (*e.g.*, fulvic acid and humic acid substances), which was observed in the foulant layers, albeit in lower abundance compared to microbial byproducts (see Figure 3A–D), can affect the dominant products of the Mn(II)–Fe oxide catalytic oxidation described above. NOM can stabilize dissolved Mn(II) and Mn(III) species in solution through complexation reactions, and, hence, inhibit Mn(III, IV) (oxy)(hydro)oxide formation.^56^ More complex interaction mechanisms have been confirmed by Ma et al. for a ternary Mn(II)–NOM–Fe(III) oxide colloidal system under oxic, alkaline (pH = 8) conditions.^56^ These include the initial catalytic oxidation of Mn(II) to Mn(III, IV) species, which in turn oxidize NOM and produce Mn(II) that is reused in the next oxidation–reduction cycle at the Fe(III) colloid surface.^56^ Further research is needed to quantify the different Mn species in the feedwater and to evaluate the relative importance of the described Mn(II)–NOM–Fe(III) oxide system.

##### 3.4.2.2 Effect of Biotic Interactions

Considering the high TCC in the feedwater and evidence of biofouling of the membrane surface (Figure 3E,F), the effect of biotic processes on Mn cycling also needs to be taken into account. We speculate that biological processes play a role in Mn cycling and accumulation on the membrane surface *via* two mechanisms (Figure 4B,C). On the one hand, the elevated Mn concentration in the membrane deposits can be explained by interactions between the dissolved ionic Mn (*i.e.*, Mn(II) and Mn (III)) and EPS (Figure 4C). Strong complexation of alginate in the presence of Ca^2+^ and Mg^2+^, attributed to the divalent bridging of deprotonated carboxylic groups on neighboring alginate molecules, has been shown to not only sequester the ions from solution but also cause significant NF membrane performance issues and up to 75% permeate flux reduction.^59,60^ Alternatively, the microorganisms in the biofoulant layer on the membrane surface could be facilitating Mn oxidation and subsequent *in situ* precipitation^49,61^ (Figure 4B). Incubation experiments simulating oxidation processes in the water column with dissolved Mn(II) have shown negligible oxidation over 1 month period in abiotic environment, compared to *t*_0.5_ ∼ 30 days under biotic conditions.^36,45^ Faster Mn oxidation times of few days were observed when the microorganisms were concentrated at higher density on particulates.^36^ In the context of DWTP, the biofouled membrane surface could act as a hot spot for Mn oxidation. This is consistent with the high levels of Mn and protein determined on the heavily fouled membrane tubes (63 mg Mn/m^2^ + 5.9 μg protein/cm^2^), with both indicators exhibiting a sharp decline and comparable values for the tubes of mid and low fouling severity: 8 mg Mn/m^2^ + 1.3 μg protein/cm^2^, and 8 mg Mn/m^2^ + 2.4 μg protein/cm^2^, respectively (Table 1). This was not the case for the iron deposits on the membrane, where concentrations declined gradually in accordance with the surface coloration: 63, 20, and 16 mg Fe/m^2^ for high, mid, and low fouling severity, respectively (Table 1). The similar trend observed in Mn and protein content on the membrane surface provides evidence that in the current system, Mn cycling is more closely linked to, and modulated by, biological processes than mineral surfaces and organic matter. Furthermore, Mn oxidation capabilities are not limited to a specific genus, and bacteria from commonly found genera such as *Bacillus*, *Pseudomona, Acinobacter, Lysobacter*, and *Leptothix*, which have previously been detected in membrane foulant layers,^26,61^ can perform this function.^62−64^ Future investigation of the biofilm microbiome *via* DNA or RNA sequence analysis would be of interest to confirm the presence of Mn-oxidizing bacteria.

#### 3.4.3 Membrane Cleaning and Recommendations

The membrane cleaning data suggest that the two DWTPs can benefit from an improved cleaning strategy, including less frequent cleaning in winter, when the membrane operation is relatively stable (Figure S2). Our data show that citric acid cleaning is ineffectual vis-à-vis biofouled membranes (Figure S2A). The cleaning agent at plant X needs to be tailored to the specific local foulants during the summer and simultaneously target biofouling and metal accumulation. In general, acidic cleaning is the preferred method for removing inorganic colloidal foulants and scalants from the membrane surface as it dissolves inorganic colloids and alleviates the build-up on the membrane.^65,66^ This could explain why citric acid cleaning is able to maintain the membrane permeance throughout the membrane lifespan for plant Y, as the fouling is mainly composed of inorganic material. However, when colloids/particulates are embedded in EPS (excreted by microorganisms),^16,67^ an appropriate biofouling control strategy is needed as well,^68^ since citric acid was shown to be inefficient in maintaining membrane performance properties for plant X. Considering the chlorine tolerance afforded by cellulose acetate membranes,^69^ chlorine cleaning could possibly help manage biofouling in the DWTPs considered in this work, but not in others equipped with polyamide membranes (which react with Cl_2_^70^). However, due to the high TOC content in the feedwater (Table S2), chlorine-based cleaning agents could promote the formation of carcinogenic disinfection byproducts (DBPs).^25,29,71^ This can be avoided by using permeate water (with lower TOC) as a solvent for the preparation of the chlorine cleaning solution. Another disadvantage of chlorination is that strong oxidizing agents (applied for biofouling control) can interact with the metals in the feed and promote their oxidation^72^ and deposition in the membrane channel. Dealing with a complex foulant layer of multiple interacting constituents is not a trivial task, and therefore, development of chemical or physical cleaning strategies for organometallic fouling control needs to be further explored.

## 4 Conclusions

In closing, seasonal variations in temperature, precipitation, metal cycling, and microbial activity at plant X led to significant permeance loss through biofouling and metal (Fe, Mn) oxide deposits. The high concentrations of Fe and Mn detected on the membrane surface and in the feedwater during the summer months are consistent with *in situ* metal remobilization from the lake sediment. Our study shows that current cleaning-in-place (CIP) protocols (consisting of citric acid) are ineffective at restoring performance, suggesting a need to develop CIP protocols aimed at biofoulants as well as the addition of a disinfecting agent to tackle high microbial counts. Our data suggest that DWTPs in temperate regions supplied by standing surface water bodies are more vulnerable (compared to flowing water bodies, such as that feeding plant Y) to future rising temperatures and the implications that these have on feedwater composition.

## Supporting Information Available

Additional materials and methods (Sections S.1–S.4); operating and environmental conditions of membrane DWTPs (Table S1); feedwater composition (Table S2); elemental analysis of foulant layers obtained *via* SEM-EDX (Table S3); membrane transport parameters (Table S4); additional membrane operating parameters as a function of time (Figure S1); membrane permeance as a function of time, with cleaning and replacement dates noted (Figure S2); correlation analyses (Figure S3); average monthly precipitation and air temperature (Figure S4); additional monthly averaged feed composition as a function of time (Figure S5); digital images and SEM micrographs of membrane samples (Figure S6); and SEM image and EDX spectra of inorganic foulant deposits (Figure S7) (PDF)
