# Supplementary material for: Analysis of Tubular NF Plants in Scotland Indicates That Summer Temperatures and Redox-Sensitive Elements Are Correlated with Membrane Biofouling and Shortened Useful Life
Source: ACS ES T Water. 2024 Oct 29;4(11):5002–12. doi: 10.1021/acsestwater.4c00630 (PMC11555670; doi:10.1021/acsestwater.4c00630)
Supplement: Supplementary file 1 — ew4c00630_si_001.pdf [file ew4c00630_si_001.pdf]

Supporting Information

Analysis of tubular NF plants in Scotland indicates that summer temperatures and redox-sensitive elements are correlated with membrane biofouling and shortened useful life

ACS ES&T Water

Desislava Filipova Davidkova[1], Margaret Catherine Graham[2], David MacLeod[3],

Santiago Romero-Vargas Castrillón[1,4,*], Andrea Joana Correia Semiao[1,*]

[1] Institute for Infrastructure and Environment, School of Engineering, The University of Edinburgh, William Rankine Building, Thomas Bayes Road, Edinburgh EH9 3FG, United Kingdom

[2] School of Geosciences, The University of Edinburgh, Crew Building, Alexander Crum Brown Road, Edinburgh EH9 3FF, United Kingdom

[3] Scottish Water, 31 Henderson Drive, Inverness, IV1 1TR United Kingdom

[4] Institute for Materials and Processes, School of Engineering, The University of Edinburgh, Sanderson Building, Robert Stevenson Road, Edinburgh EH9 3FB, United Kingdom

* Corresponding authors: Santiago@ed.ac.uk; Andrea.Semiao@ed.ac.uk

Supporting methods and materials

S.1. Osmotic pressure calculations

The osmotic pressure (π) of the feed water was calculated from the conductivity data available for plant Y, which ranged between 30 and 168 μS cm^{-1} (Table S2) and was typical for surface freshwater[1–3]. We calculated π using the minimum and maximum conductivity values to provide a range of osmotic pressures. To compute π, the conductivity is first transformed into total dissolved solids (TDS) using the following correlation[4]:

$$TDS = k \times EC$$

where *TDS* is total dissolved solids concentration [mg/L], *EC* is the electrical conductivity [μS cm^{-1}] and *k* is a proportionality factor (assumed to be 0.55 for freshwater[4]). The osmotic pressure is then obtained from *TDS* by the following approximation[5]:

$$\pi = \frac{TDS}{1318.57}$$

with π in bar. For the range of conductivity values, we find 0.01 bar < π < 0.07 bar, showing that $\pi \ll P_{inlet}$ (Fig. 1 B and E), which justifies the omission of the π term when calculating the TMP.

S.2. Feed water quality parameters

The feed water parameters were measured in Scottish Water Laboratories using analytical protocols, facilities and materials approved by the United Kingdom Accreditation Service (UKAS). In brief, the total organic carbon (TOC) was determined as non-purgeable organic carbon (NPOC) using a total organic carbon analyser. The feed water elemental composition (Al, Fe, Mn) was quantified using inductively coupled plasma-mass spectrometry (ICP-MS) after overnight digestion of the feed water samples with 1% v/v nitric acid (Aristar® grade) at 80 °C. The results are denoted as the total (i.e., dissolved + suspended particulate) elemental concentrations in the water samples. The *E. coli* and coliform colony forming units (CFUs) were counted using the Colilert-18 method (ISO 9308-2:2012). The total cell counts (TCC) were quantified using flow cytometry. Conductivity and pH were also measured using calibrated handheld devices.

Statistical analyses were performed using R (ver. 4.2.1)[6]. For each water quality parameter, the minimum, maximum and mean value, along with the standard deviation (sd), were calculated. Furthermore, the monthly-averaged value for each water quality parameter was computed and plotted over time to provide information about seasonal trends in feed water composition. Spearman's rank correlation test was chosen to investigate the relationship between different feed and operating parameters as this non-parametric statistical method is suitable for both normally and non-normally distributed data (as was commonly found in the data reported herein).

S.3. Membrane autopsy protocols

Used membrane samples from each DWTP were provided by Scottish Water for further laboratory analysis. The membranes at plant X were replaced on 21/01/2022 and at plant Y on 06/12/2022. After

their removal from the modules, individual membrane tubes were placed in sealed plastic bags with a small amount of 0.25% v/v sodium metabisulphite and stored at 4 °C in darkness to prevent further biological growth. The membrane tubes were cut lengthwise, and the active layer side was photographed. Pristine CA202 membranes were used in the characterisation analyses as negative controls.

All solutions used in the laboratory analyses were prepared using ultrapure (UP)-grade water (18.2 MΩ cm resistivity) (Alto™ I Ultrapure water system, Triple Red Ltd.). Before use, glassware was acid-washed and rinsed with UP water. The organic foulants on the membrane surface were extracted using UP water extraction[7,8] as follows: membrane cut-outs (8 cm^2) were placed in centrifuge tubes and suspended in 14 mL of UP water. The centrifuge tubes were bath-sonicated for 2 minutes, followed by vortex mixing for 5 seconds. This cycle was repeated 3 times. The supernatants were used for TOC analysis (Shimadzu, Japan), UV-visible spectrophotometric analysis and fluorescence emission-excitation matrix (FEEM) analysis (Cary Eclipse, Variant). The TOC analysis was done in triplicate for each membrane.

Protein extraction and analysis: Protein extraction was performed following a method adapted from Bar-Zeev and Elimelech[9]. The membrane subsections (4 cm^2) were suspended in 1.2 mL ultrapure (UP) water in microcentrifuge tubes (Beckman Coulter, UK) and bath sonicated for 60 minutes. The membranes were removed and the tubes were centrifuged at 24,000 × g for 25 minutes at 4 °C using an ultracentrifuge (Optima Beckman Coulter, UK) equipped with the TLA-55 rotor (Beckman Coulter, UK). The supernatant was discarded and the pellet was re-suspended in 1 mL 1X Lauber buffer (50 mM HEPES (pH 7.3), 100 mM NaCl, 10% sucrose, and 0.1% 3-[(3-cholamidopropyl)-dimethylammonio]-1 propanesulfonate) and vortex-mixed for 30 seconds. The samples were subsequently centrifuged at 2,730 × g for 1 minute and probe sonicated on ice (three 30-second cycles). Finally, the tubes were centrifuged again at 24,000 × g for 10 minutes at 4 °C. The total protein biomass extracted from the fouled membranes

was quantified colorimetrically using the Pierce™ Bicinchoninic acid (BCA) protein assay (Thermo Scientific, UK) and ultraviolet-visible spectroscopy (Cary 100 Varian, UK), measuring absorbance at 562 nm. The extraction and subsequent assay was performed in triplicate for each membrane tube.

FEEM analysis: For the fluorescence excitation-emission matrix (FEEM) analysis, the supernatants from the UP water extraction were placed in a quartz cuvette. The spectra were obtained with a fluorescence spectrophotometer (Cary Eclipse Variant, UK) with excitation and emission wavelengths set to 220 – 450 nm and 250 – 550 nm, respectively and a scan step of 5 nm. Further data processing required collecting fluorescence spectra of blank samples (UP water) and the UV-visible light absorbance (200 – 800 nm) of all samples using a UV-vis spectrophotometer (100 Cary Varian, UK). The fluorescence data processing was done in R (version 4.2.1) using the eemR package[10]. This included blank subtraction, removal of 1st- and 2nd- order Raman and Rayleigh scattering and inner-filter correction.

Acid digestion and elemental analysis: Subsections (8 cm^2) of the pristine and decommissioned membrane samples were cut, placed in aluminium tins and oven dried at 40 °C for 24 hours. The samples were transferred to 100-mL beakers, covered with watch glasses and placed in a muffle furnace at 450 °C for 4 hours to remove the organic carbon from the samples. Next, 15 mL of 37% w/w HCl (Acros Chemicals, UK) and 5 mL of 69% w/w HNO_3 (Fisher Scientific, UK) were added to each beaker. These were covered with the watch glasses, transferred onto a hot plate, and left to reflux for 2 hours at 120 – 140 °C. The watch glasses were uncovered and the samples were boiled down to a small volume (1 – 2 mL). The samples were carefully transferred to 25-mL volumetric flasks, filtered through a Whatman No. 41 ashless filter paper and made up to volume (25 mL) by adding 2% w/w HNO_3. The samples were stored at 4 °C until the elemental analysis was performed by inductively coupled plasma mass spectrometry (Agilent 7900, UK). As part of the quality control, the digestion was done in triplicate for each membrane tube; procedural blanks (15 mL of 37% w/w HCl and 5 mL of 69% w/w HNO_3) were

analysed to account for any contamination and samples of standard elemental concentration were analysed to check for instrument drift. The final elemental content of the analysed samples was blank corrected, expressed per membrane area and averaged from the three replicates.

Cryo-SEM-EDX sample preparation and analysis: Pristine and decommissioned membrane samples were cut and glued (using equal parts of colloidal graphite (G303, Aquadag®) and OCT compound (Scigen®)) to a copper stub. The samples were dipped in slushed nitrogen, sublimed for 5 minutes (T = -90 °C, $P \approx 10^{-7}$ mbar) and sputter coated with Pt to ensure high conductivity of the sample. During the imaging the probe current used was 50 – 100 pA and the acceleration voltage was 5 – 20 kV.

S.4. Membrane characterisation protocols

The parameters in Table S4 were determined using a custom-built crossflow set-up (specifications are available in our previous publication[11]). Briefly, the molecular weight cutoff (MWCO) (defined as the smallest molecular weight of the compound which is 90% rejected by the membrane) of the pristine CA202 membrane was determined by measuring the TOC rejection of polyethylene glycol (PEG, 600 – 10000 g/mol) at transmembrane pressure TMP = 2.5 bar, permeate flux J = 10.18 ± 0.7 LMH, crossflow velocity u = 2.04 m/s (Re = 2580), and temperature T = 20 °C. The TOC content of the feed and the permeate were determined using a TOC analyser (Shimadzu, Japan).

The permeance (A_0) of the pristine CA202 membrane was determined by measuring the water volumetric flow rate through the membrane over time (10 minutes) using a flow sensor (SLF3S-1300F, Sensirion, Switzerland). The filtration conditions were TMP = 10 bar, u = 0.55 m/s (Re = 920), pH = 5.5 and T = 20 °C.

The salt rejection of the pristine CA202 membrane was determined by measuring the feed and permeate conductivity with either a $CaCl_2$ or NaCl feed solution using a hand-held conductivity meter (Con6+, Oakton USA). The filtration conditions were J = 100 LMH, u = 0.55 m/s (Re = 920), pH = 5.5, I = 1 mM and T = 20 °C.

Supporting results

Table S1. Operating and environmental conditions of membrane drinking water treatment plants.

	Plant	
	**Plant X**	**Plant Y**
**Operating parameters [a]**		
**Membrane**	Tubular CA202 (PCI, Poland)	
**Membrane material**	Cellulose acetate	
**membrane area [m^2]**	354	249.6
**Pre-treatment**	2-mm screening mesh	
**Cleaning**	Physical: foam ball scouring (every 4 – 6 h)<br>Chemical: citric acid cleaning (every 2 – 5 months)	
**Environmental parameters [b]**		
**Water source type**	Lake (low alkalinity)	Stream
**Loch surface area [ha]**	5	-
**Mean depth [m]**	4.5	-
**Elevation [m]**	22	-
**Dominant catchment landcover**	Grassland (with dairy farming activity in the area)	Upland catchment: mixed grassland and heather moor

[a] data provided by Scottish Water; [b] data available from UK Centre for Ecology and Hydrology[12].

Table S2. Feed water composition for two NF DWTPs in Scotland over a 4.5-year time period. NA – not available.

Parameter	unit	Plant X			Plant Y		
		**mean ± sd**	**Min**	**Max**	**mean ± sd**	**Min**	**Max**
Fe	μg L^{-1}	322 ± 179	71	821	120 ± 178	7	2,874
Mn	μg L^{-1}	57 ± 85	4	643	4.9 ± 11.2	1	209
Al	μg L^{-1}	19 ± 12	8	58	44 ± 60	8	696
TOC	mg L^{-1}	7.0 ± 1.6	0.2	11.2	1.9 ± 1.9	0.2	14
pH		-	7.2	8.0	-	7.1	8.0
Conductivity	μS cm^{-1}	NA			127 ± 24	30	168
Coliforms	CFU/100 mL	332 ± 942	2	9,200	221 ± 274	10	2,200
E. coli	CFU/100 mL	22 ± 69	0	1,000	90 ± 190	0	1,000
TCC	× 10^3 mL^{-1}	3,615 ± 2,060	233	10,112	796 ± 2,516	0	31,528

Table S3. Element weight percentage (wt%) for four spectra collected from a heavily fouled membrane from pant X using SEM-EDX.

Element [wt%]	Spectrum 1	Spectrum 2	Spectrum 3	Spectrum 4
O	49.3	54.8	81	98.5
Mn	35.8	30.3	-	-
Fe	6.5	6.4	-	-
Ca	3.5	3.1	13.3	-
C	2.5	2.3	-	-
Al	1.1	1.3	3	1.5
S	1	1.4	2.4	-
Mg	0.3	0.3	0.4	-
Na	0.1	0.1	-	-

Note: the weight of the coating element (Pt) has been subtracted and the total weight of the remaining elements has been adjusted to 100%.

Table S4. Permeance and rejection coefficients of membranes used in plants X and Y.

Membrane	Active layer material	MWCO [Da]	A_0 [LMH/bar][a]	NaCl rejection [%][a]	$CaCl_2$ rejection [%][a]
CA202	Cellulose Acetate	2500 - 5200 [a]	5.0 ± 0.5	9.2 ± 1.6	6.2 ± 1.0

[a] Values from this study; LMH – L m^{-2} h^{-1}; MWCO – molecular weight cut-off; A_0 – UP water permeance.

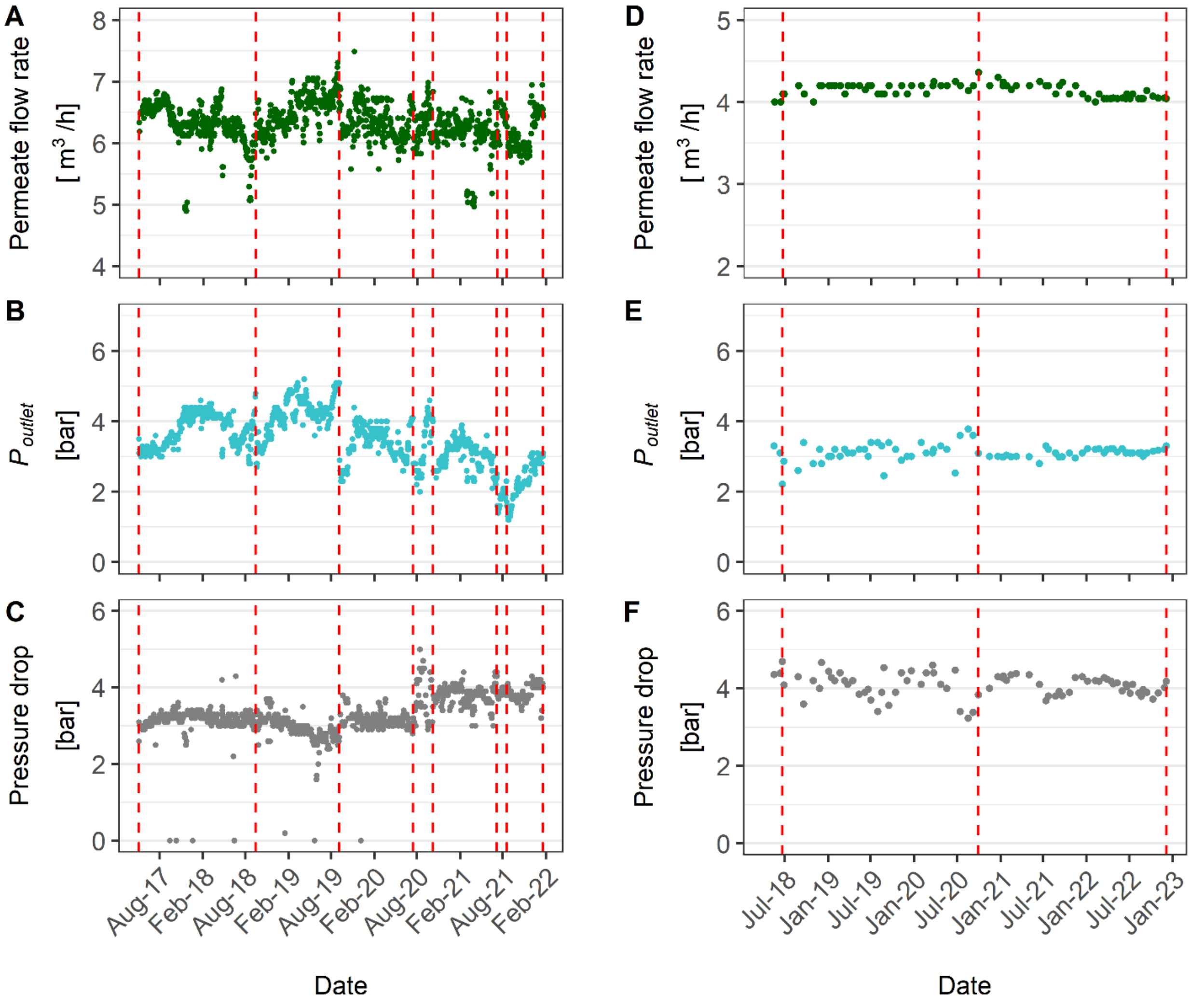

Figure S1. Membrane operating parameters over time for Plant X (A – C) and Plant Y (D – F) including permeate flow rate, outlet pressure (P_{outlet}) measured at the retentate side of the membrane channel and the feed channel pressure drop ($PD = P_{inlet} - P_{outlet}$). The red dashed vertical lines indicate dates of membrane replacement.

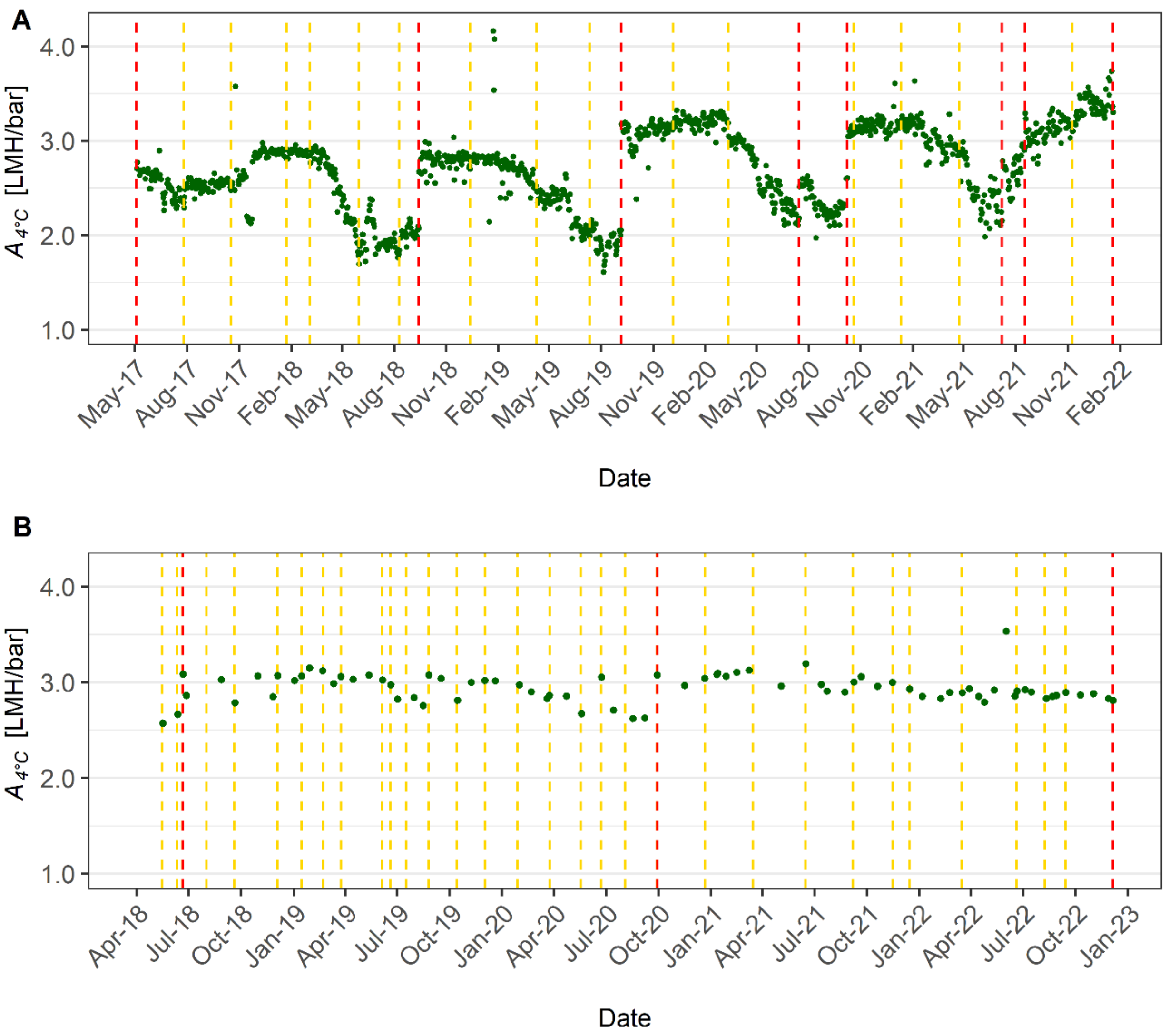

Figure S2. Membrane permeance ($A_{4°C}$) over time for Plant X (A) and Plant Y (B). The red dashed vertical lines indicate dates of membrane replacement and the yellow dashed vertical lines indicate membrane chemical (citric acid) cleaning.

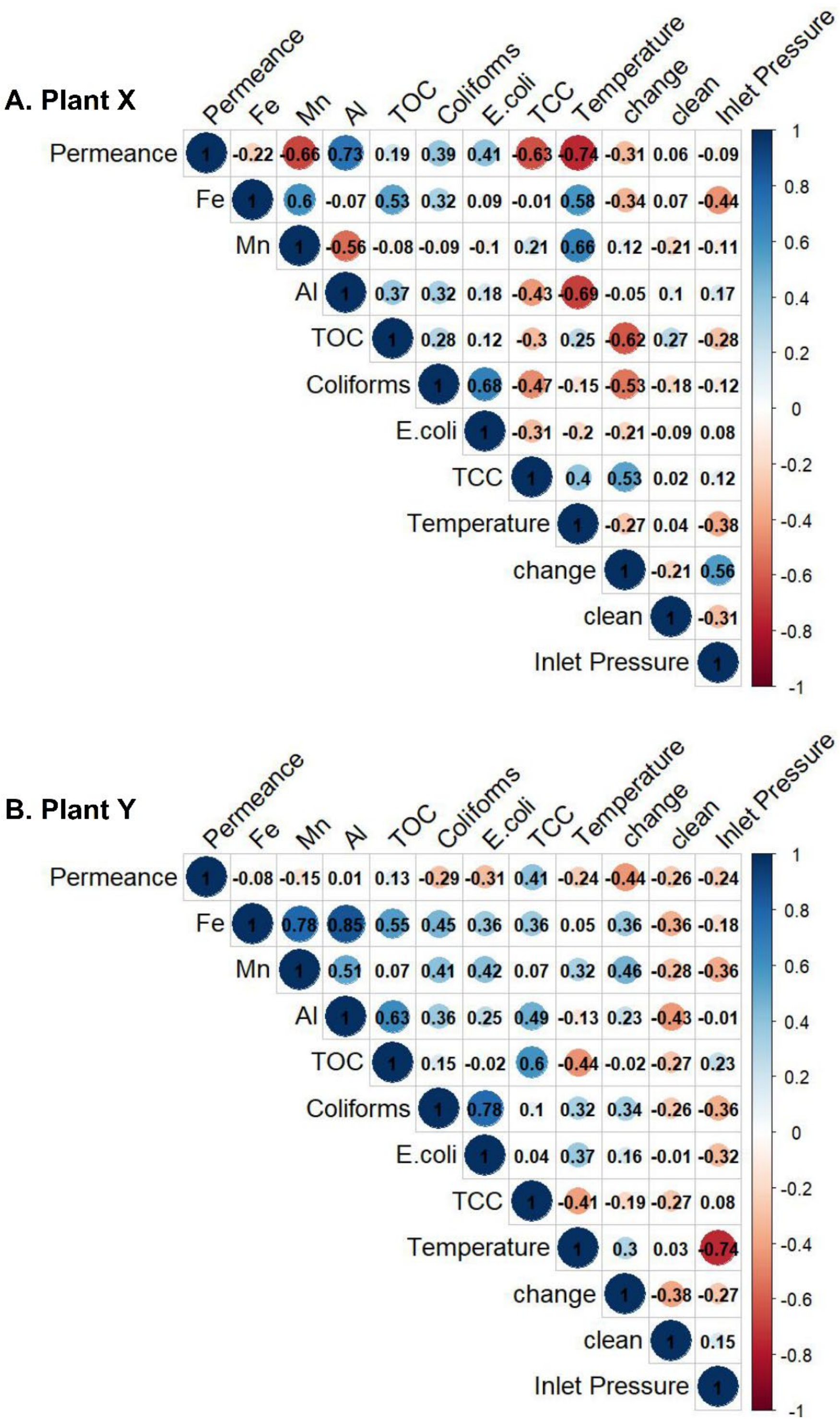

Figure S3. Correlation matrices showing the Spearman's correlation coefficient (*r*) between various feed water quality and operating parameters for Plant X (A) and Plant Y (B); Note: clean – days since last membrane cleaning; change – days since last membrane change.

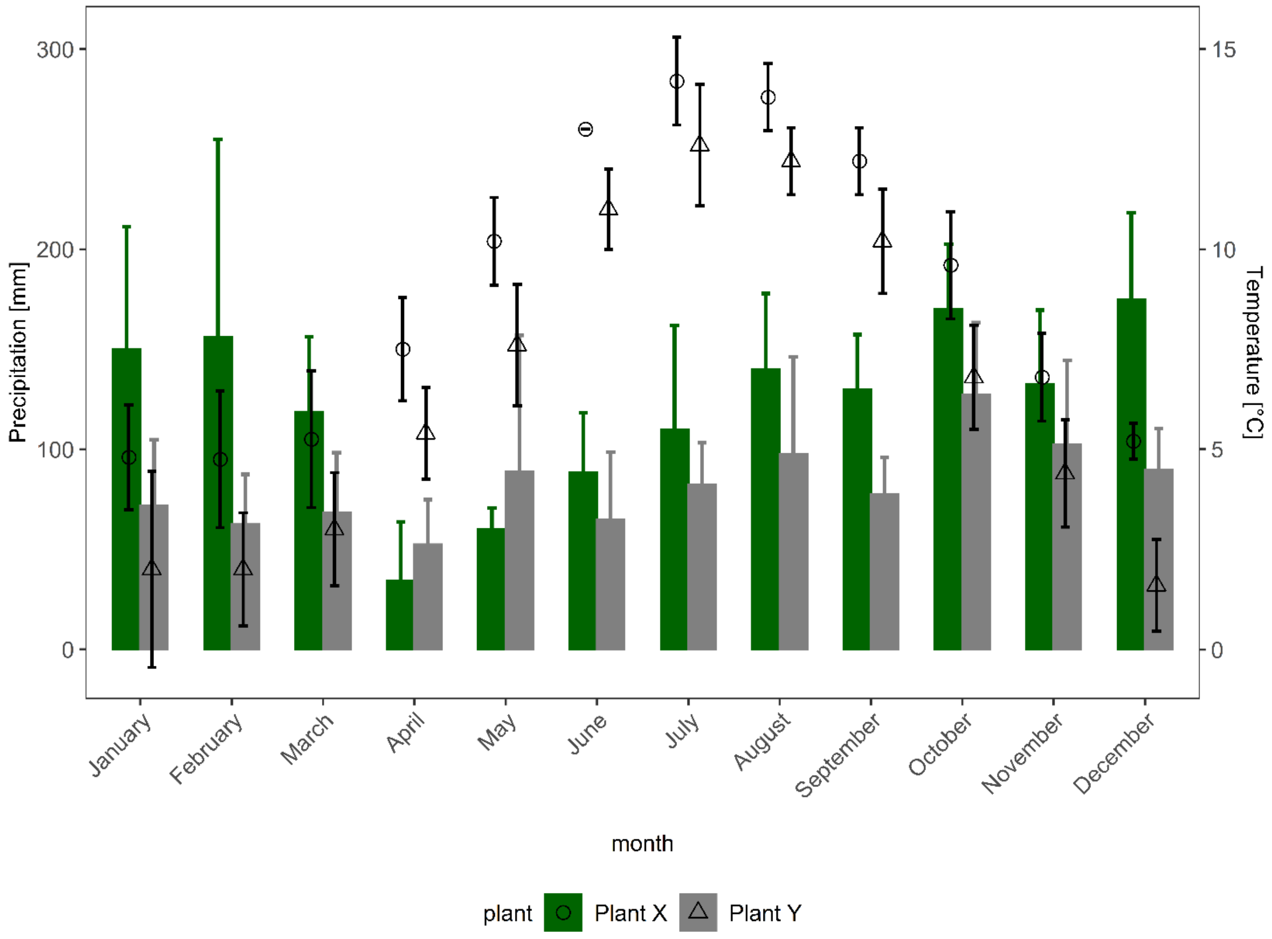

Figure S4. Average monthly precipitation levels (bar chart) and air temperatures (calculated from daily mean temperatures) (scatter plot; right y-axis) for Plant X (May 2017 – Jan 2022) and Plant Y (April 2018 – Dec 2022); error bars indicate standard deviation around the mean (data from [13]).

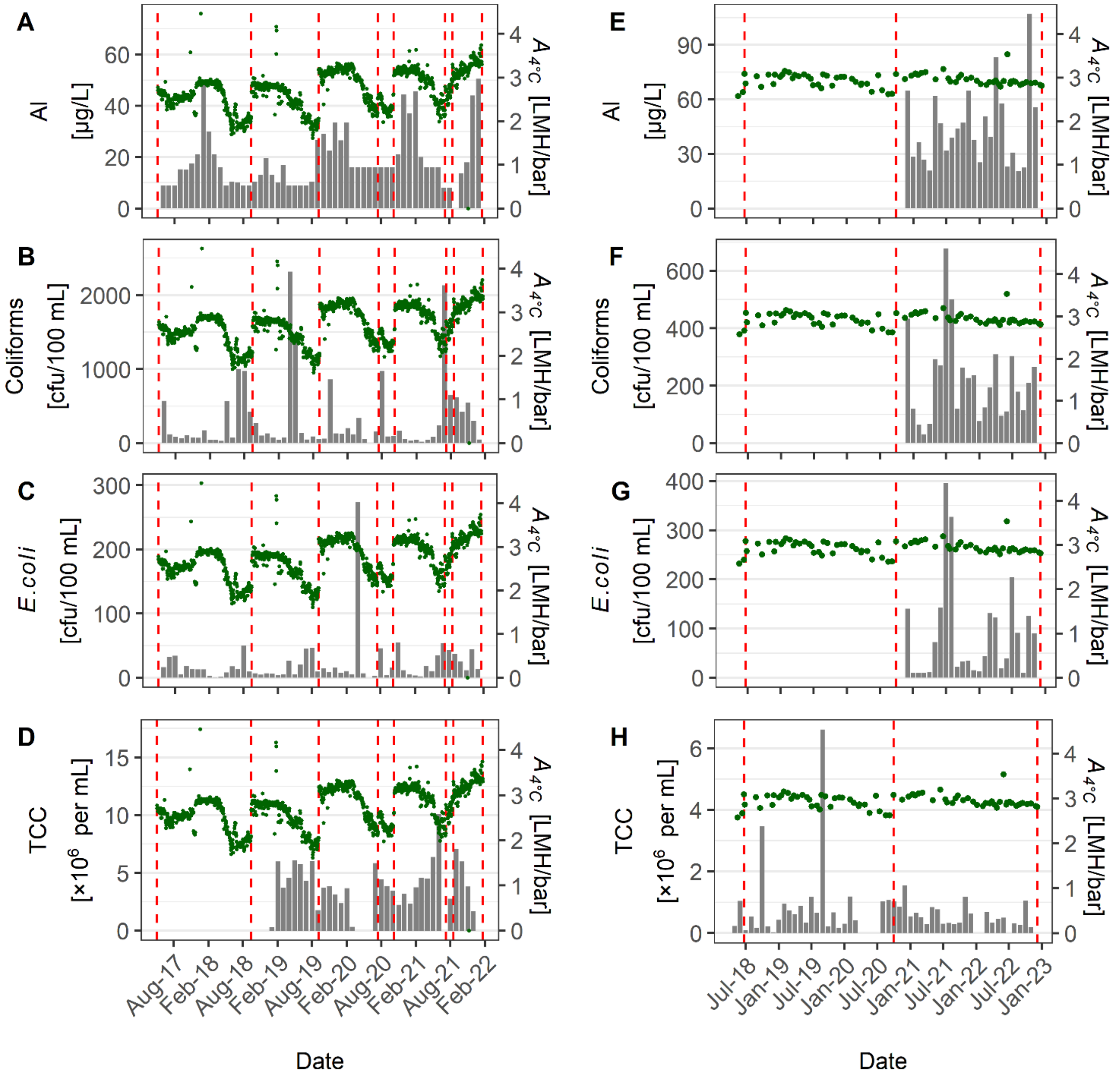

Figure S5. Monthly-averaged feed quality parameters (bar plot) over time overlaid with permeance (scatter plot; right y-axis) for Plant X (A – D) and Plant Y (E – H). Note: the scales of the y-axis differ for Plant X and Y.

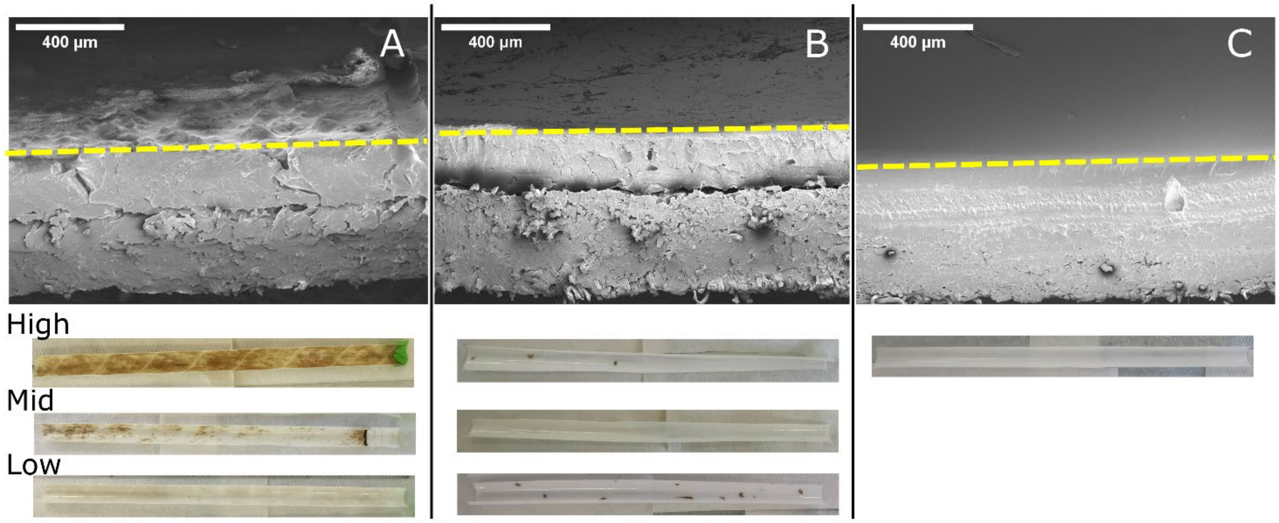

Figure S6. Visual inspection and surface imaging by SEM of the NF membrane tubes from plant X (A) and plant Y (B) and a pristine tubular CA202 membrane (C). Samples were imaged at an angle of 15 – 30° to capture both the cross-sectional area and the top sample surface (the yellow dashed lines indicate the approximate location of the boundary between the former and the latter).

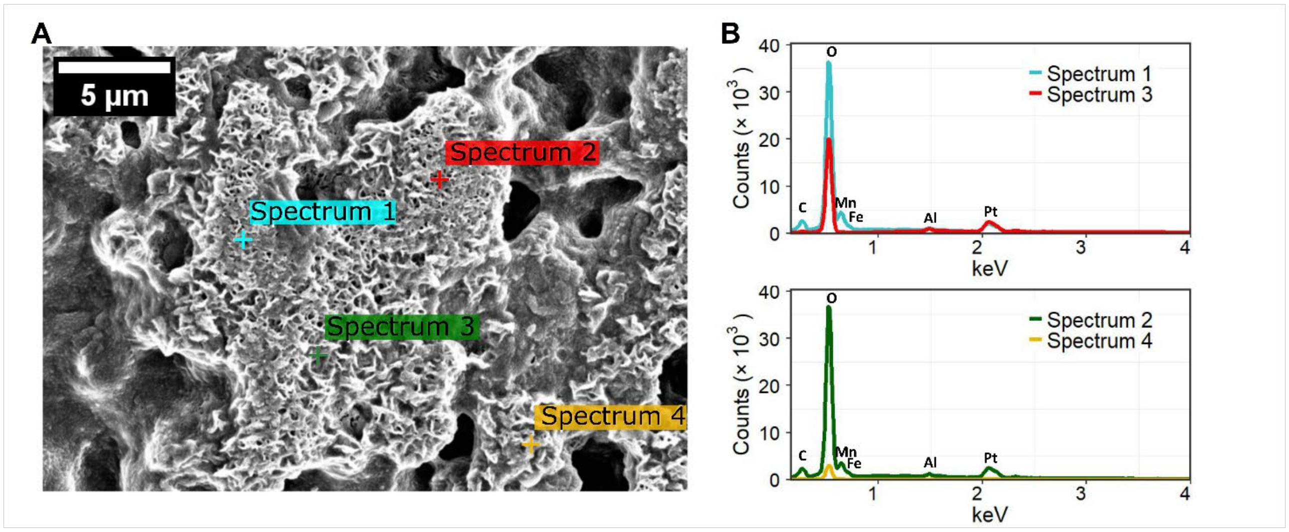

Figure S7. SEM image of inorganic deposits on tubular membranes from plant X (A); EDX spectra of sampled areas (B).

References

(1) Bowen, W. R.; Doneva, T. A.; Yin, H. B. Separation of Humic Acid from a Model Surface Water with PSU/SPEEK Blend UF/NF Membranes. *J Memb Sci* **2002**, *206* (1–2), 417–429. https://doi.org/10.1016/S0376-7388(01)00786-4.

(2) Valdivia-Garcia, M.; Weir, P.; Graham, D. W.; Werner, D. Predicted Impact of Climate Change on Trihalomethanes Formation in Drinking Water Treatment. *Sci Rep* **2019**, *9* (1), 1–10. https://doi.org/10.1038/s41598-019-46238-0.

(3) Valdivia-Garcia, M.; Weir, P.; Frogbrook, Z.; Graham, D. W.; Werner, D. Climatic, Geographic and Operational Determinants of Trihalomethanes (THMs) in Drinking Water Systems. *Sci Rep* **2019**, *6* (1), 1–12. https://doi.org/10.1038/srep35027.

(4) Rusydi, A. F. Correlation between Conductivity and Total Dissolved Solid in Various Type of Water: A Review. *IOP Conf Ser Earth Environ Sci* **2018**, *118* (1). https://doi.org/10.1088/1755-1315/118/1/012019.

(5) El-Dessouky, H. T.; Ettouney, H. M. Reverse Osmosis. In *Fundamentals of Salt Water Desalination*; Elsevier Science B.V., 2002; pp 410–437. https://doi.org/10.1016/C2016-0-00580-6.

(6) R Core Team. R: A Language and Environment for Statistical Computing. Vienna, Austria 2022.

(7) Beyer, F.; Rietman, B. M.; Zwijnenburg, A.; van den Brink, P.; Vrouwenvelder, J. S.; Jarzembowska, M.; Laurinonyte, J.; Stams, A. J. M.; Plugge, C. M. Long-Term Performance and Fouling Analysis of Full-Scale Direct Nanofiltration (NF) Installations Treating Anoxic Groundwater. *J Memb Sci* **2014**, *468*, 339–348. https://doi.org/10.1016/j.memsci.2014.06.004.

(8) Vrouwenvelder, J. S.; Manolarakis, S. A.; van der Hoek, J. P.; van Paassen, J. A. M.; van der Meer, W. G. J.; van Agtmaal, J. M. C.; Prummel, H. D. M.; Kruithof, J. C.; van Loosdrecht, M. C. M. Quantitative Biofouling Diagnosis in Full Scale Nanofiltration and Reverse Osmosis Installations. *Water Res* **2008**, *42* (19), 4856–4868. https://doi.org/10.1016/j.watres.2008.09.002.

(9) Bar-Zeev, E.; Elimelech, M. Reverse Osmosis Biofilm Dispersal by Osmotic Back-Flushing: Cleaning via Substratum Perforation. *Environ. Sci. Technol. Lett* **2014**, *1*, 17.

(10) Massicotte, P. *EemR: Tools for Pre-Processing Emission-Excitation-Matrix (EEM) Fluorescence Data*; 2019.

(11) Davidkova, D. F.; Graham, M.; Romero-Vargas Castrillón, S.; Semiao, A. Influence of Colloidal Iron Oxide and Natural Organic Matter Fouling on Nanofiltration Membrane Performance: Role of Feed Composition and Membrane Properties. *Environ Sci (Camb)* **2023**. https://doi.org/10.1039/D3EW00495C.

(12) UK CEH. *UK Lakes Portal*. https://eip.ceh.ac.uk/apps/lakes/.

(13) UK Met Office. *UK actual and anomaly maps*. https://www.metoffice.gov.uk/research/climate/maps-and-data/uk-actual-and-anomaly-maps.
